# Intravenous Lobular Capillary Hemangioma: A Case Report and Proposal for New Cutaneous Tender Tumor Differential Diagnosis Acronym

**DOI:** 10.7759/cureus.28030

**Published:** 2022-08-15

**Authors:** Brynn Sargent, Suzanne W Birmingham, Hadas Skupsky

**Affiliations:** 1 Dermatology, University of California Irvine, Orange, USA; 2 Dermatology, University of California San Francisco, San Francisco, USA; 3 Dermatopathology, University of California Irvine, Orange, USA

**Keywords:** intravenous lobular capillary hemangioma, pyogenic granuloma, painful skin lesions, subcutaneous nodule, dermal nodule, cutaneous tumor

## Abstract

Here, we report a case of a 70-year-old female who presented with a slowly enlarging tender nodule on the right forearm for several months. Physical examination showed a faintly blue-tinged freely mobile subcutaneous nodule. Excision was complicated by greater than expected bleeding and revealed an unexpected intravenous mass. Histopathology demonstrated capillary lobules separated by fibrous septae within a vein, consistent with intravenous lobular capillary hemangioma (IVLCH). IVLCH is a rare benign capillary proliferation of unclear etiology. Excision is typically curative and relieves any pain and discomfort the patient might be experiencing. With the addition of IVLCH, we respectfully propose a new acronym for the differential diagnosis of cutaneous tender tumors: intravenous lobular capillary hemangioma, foreign body (reaction), hidradenoma, osteoma cutis, glomus tumor, scar, fibromyxoma, leiomyosarcoma, eccrine angiomatous hamartoma, Dercum’s disease (adiposis dolorosa), piezogenic pedal papule, eccrine spiradenoma, neurilemmoma (schwannoma), calcinosis cutis, angioendotheliomatosis, leiomyoma, metastases, angiolipoma, neuroma, dermatofibroma, granular cell tumor, endometriosis, thrombus, blue rubber bleb nevus, angioma, chondrodermatitis nodularis helicis, and keloid (“IF HOGS FLED PEN, CALM AND GET BACK”). Future additions to the cutaneous tender tumor differential diagnosis may require creative additions and rearrangements to this acronym. However, continual updates will allow it to serve both clinicians and pathologists alike as a comprehensive representation of etiologies to consider for cutaneous tender tumors.

## Introduction

Subcutaneous and dermal nodules can represent a wide variety of different lesions. In some patients, clinical history or specific features on examination may favor one disease process over another. At other times, clinical features can be relatively nonspecific, and diagnosis requires tissue biopsy and histopathological evaluation. For painful lesions, the differential diagnosis is somewhat more limited and therefore can help narrow the possibilities prior to microscopic evaluation. The cutaneous tender tumor differential diagnosis has been explored previously [1-4]. Most recently, after a thorough review of the literature, Cohen et al. created a catchy mnemonic loosely based on the popular children’s novel, Charlotte’s Web: “CALM HOGS FLED PENS AND GET BACK” [4]. This acronym covers many causes of cutaneous tender tumors including, in order: calcinosis cutis, angioendotheliomatosis, leiomyoma, metastases, hidradenoma, osteoma cutis, glomus tumor, scar, fibromyxoma, leiomyosarcoma, eccrine angiomatous hamartoma, Dercum’s disease, piezogenic pedal papule, eccrine spiradenoma, neurilemmoma, something else, angiolipoma, neuroma, dermatofibroma, granular cell tumor, endometriosis, thrombus, blue rubber bleb nevus, angioma, chondrodermatitis nodularis helicis, and keloid [4]. Here, we present a case of a cutaneous tender tumor not specifically covered by this previous acronym, and therefore, we respectfully propose a new all-encompassing mnemonic.

## Case presentation

**Figure 1:**
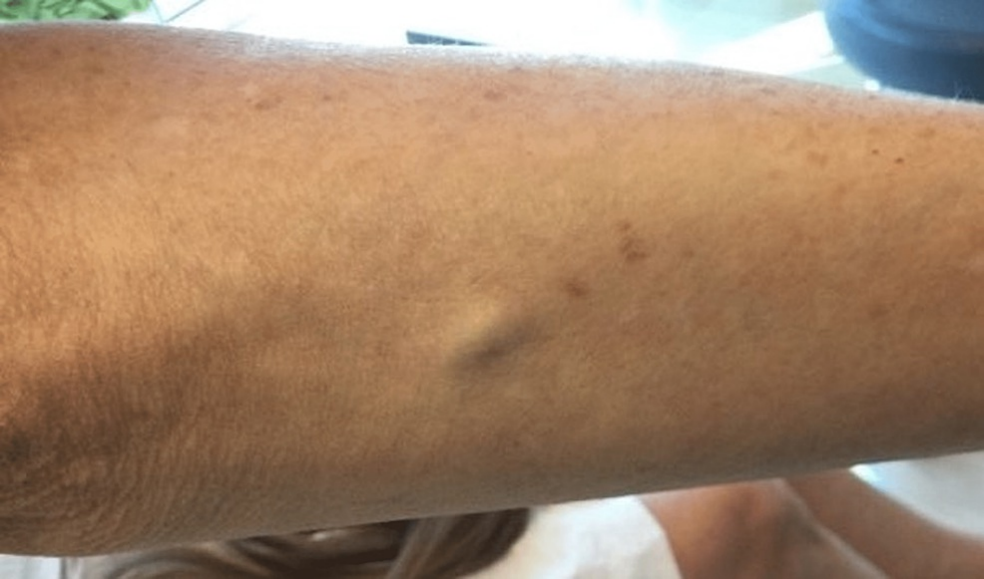
Clinical presentation Freely mobile subcutaneous nodule on the right proximal dorsal forearm

A 70-year-old female with a history of previously treated non-melanoma skin cancer presented with a slowly enlarging nodule on the right forearm that presented for several months. The nodule was tender on palpation, but the patient denied pruritus or bleeding. The patient could not identify any inciting event or local trauma nor any alleviating measures for the pain. Examination revealed a 1.5 × 1 cm, freely mobile, faintly blue-tinged subcutaneous nodule on the right proximal dorsal forearm (Figure 1).

**Figure 2:**
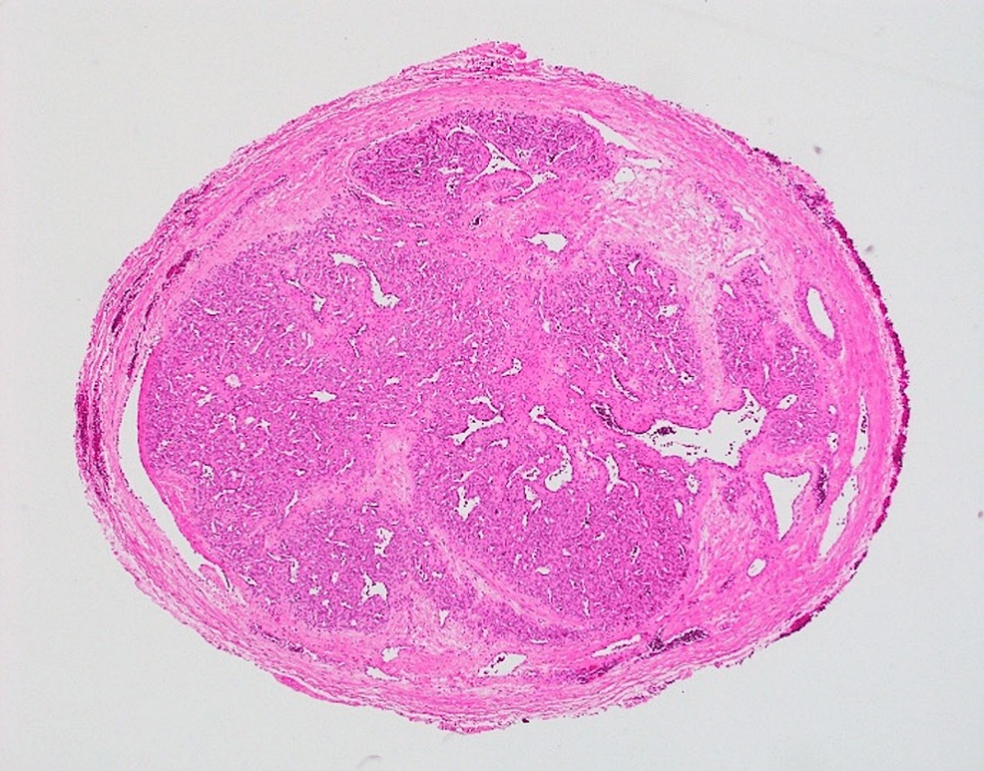
Histopathological findings Well-circumscribed nodular proliferation of tightly packed lobules of capillaries separated by fibrous septae within a vein (H&E: 40×)

Excisional biopsy was notable for heavier than expected bleeding and revealed an intravenous mass. Histopathology demonstrated a well-circumscribed, nodular proliferation of tightly packed capillary lobules separated by fibrous septae within an endothelial-lined space (Figure 2).

A diagnosis of intravenous lobular capillary hemangioma (IVLCH) was made given the intraluminal location and microscopic features. No evidence of recurrence was noted at four months postoperatively.

## Discussion

IVLCH was first described in an 18-case series by Cooper et al. in 1979 as an intraluminal polyp attached to the wall of a vein by a fibrovascular stalk [5]. IVLCHs are a relatively rare cutaneous tumor with fewer than 100 cases reported in the literature [6]. The exact etiology of IVLCH remains unclear, although some have suggested that they represent neoplastic proliferation of the vasa vasorum within the vein wall or a hyperproliferative response to local trauma or infection [7]. Clinically, these lesions present as painful or painless dermal or subcutaneous nodules without significant surface change, most commonly involving the head and neck or upper extremities [6]. Given these nonspecific features, the diagnosis is often made histopathologically. IVLCH is a completely benign lesion, and excision is typically curative, relieving any associated pain or discomfort experienced by the patient.

The cutaneous tender tumor differential is broad and has been explored at length by Cohen et al. with their clever creation of an acronym based on the popular children’s book, Charlotte’s Web [4]. However, in the most recent iteration, “CALM HOGS FLED PENS AND GET BACK,” an “S” was added to cover the category “something else” [4]. This change was meant to prevent the acronym from continuing to grow longer and allow all future additions to the cutaneous tender tumor differential to be included under this heading. While we understand the challenges presented by an increasingly unwieldy differential acronym, such simplification makes it difficult for users to recall all components within this category. In effect, this reduces the utility of the acronym, which would best serve users as an all-encompassing mnemonic. Therefore, we respectfully propose an updated acronym for cutaneous tender tumor differential: “IF HOGS FLED PEN, CALM AND GET BACK” (Table 1).

**Table 1:** Acronym for cutaneous tender tumor differential diagnosis Notes: Previous acronym presented by Cohen et al. (2020) [4] *Addition made to the previous acronym **Removal from the previous acronym

Previous acronym		Newly proposed acronym	
C	Calcinosis cutis	I*	Intravenous lobular capillary hemangioma
A	Angioendotheliomatosis	F*	Foreign body (reaction)
L	Leiomyoma		
M	Metastases	H	Hidradenoma
		O	Osteoma cutis
H	Hidradenoma	G	Glomus tumor
O	Osteoma cutis	S	Scar
G	Glomus tumor		
S	Scar	F	Fibromyxoma
		L	Leiomyosarcoma
F	Fibromyxoma	E	Eccrine angiomatous hamartoma
L	Leiomyosarcoma	D	Dercum’s disease (adiposis dolorosa)
E	Eccrine angiomatous hamartoma		
D	Dercum’s disease (adiposis dolorosa)	P	Piezogenic pedal papule
		E	Eccrine spiradenoma
P	Piezogenic pedal papule	N	Neurilemmoma (schwannoma)
E	Eccrine spiradenoma		
N	Neurilemmoma (schwannoma)	C	Calcinosis cutis
S**	Something else	A	Angioendotheliomatosis
		L	Leiomyoma
A	Angiolipoma	M	Metastases
N	Neuroma		
D	Dermatofibroma	A	Angiolipoma
		N	Neuroma
G	Granular cell tumor	D	Dermatofibroma
E	Endometriosis		
T	Thrombus	G	Granular cell tumor
		E	Endometriosis
B	Blue rubber bleb nevus	T	Thrombus
A	Angioma		
C	Chondrodermatitis nodularis helicis	B	Blue rubber bleb nevus
K	Keloid	A	Angioma
		C	Chondrodermatitis nodularis helicis
		K	Keloid

This iteration removes the “S” for “something else” and adds in “I” for “IVLCH” as presented here and “F” for “foreign body (reaction),” which Cohen et al. originally included under the “something else” category in their 2020 report [4]. While future additions to this differential will require creative additions or rearrangements of the acronym, it will continue to provide a comprehensive and exhaustive list of likely etiologies for clinicians and pathologists alike.

## Conclusions

Intravenous lobular capillary hemangioma (IVLCH) is a relatively uncommon but important consideration in the differential diagnosis of cutaneous tender tumors. Although they are completely benign, removal of IVLCHs can be complicated by greater than expected bleeding because of their intraluminal location. Given the importance of considering IVLCH in patients with cutaneous tender tumors, we respectfully propose a new acronym for this differential diagnosis: “IF HOGS FLED PEN, CALM AND GET BACK.” New etiologies will continue to require creative additions to this mnemonic, but it will serve as a comprehensive differential diagnosis for clinicians and pathologists alike.
